# Impact of local anesthetics on epigenetics in cancer

**DOI:** 10.3389/fonc.2022.849895

**Published:** 2022-08-30

**Authors:** Lucillia Bezu, Oliver Kepp, Guido Kroemer

**Affiliations:** ^1^ Equipe Labellisée Par La Ligue Contre Le Cancer, Université de Paris, Sorbonne Université, INSERM UMR1138, Centre de Recherche des Cordeliers, Institut Universitaire de France, Paris, France; ^2^ Metabolomics and Cell Biology Platforms, Gustave Roussy Cancer Campus, Université Paris Saclay, Villejuif, France; ^3^ Service d’Anesthésie Gustave Roussy Cancer Campus, Villejuif, France; ^4^ Pôle de Biologie, Hôpital Européen Georges Pompidou, AP-HP, Paris, France

**Keywords:** local anesthetics, epigenetic, cancer, demethylation, miRNA

## Abstract

Defective silencing of tumor suppressor genes through epigenetic alterations contributes to oncogenesis by perturbing cell cycle regulation, DNA repair or cell death mechanisms. Reversal of such epigenetic changes including DNA hypermethylation provides a promising anticancer strategy. Until now, the nucleoside derivatives 5-azacytidine and decitabine are the sole DNA methyltransferase (DNMT) inhibitors approved by the FDA for the treatment of specific hematological cancers. Nevertheless, due to their nucleoside structure, these inhibitors directly incorporate into DNA, which leads to severe side effects and compromises genomic stability. Much emphasis has been placed on the development of less toxic epigenetic modifiers. Recently, several preclinical studies demonstrated the potent epigenetic effects of local anesthetics, which are routinely used during primary tumor resection to relief surgical pain. These non-nucleoside molecules inhibit DNMT activity, affect the expression of micro-RNAs and repress histone acetylation, thus exerting cytotoxic effects on malignant cells. The in-depth mechanistic comprehension of these epigenetic effects might promote the use of local anesthetics as anticancer drugs.

## Introduction

### Epigenetic alterations and cancer

Epigenetic alterations are common molecular hallmarks of most cancers (1). In normal cells, epigenetic changes are fundamental for the control of gene expression, for the maintenance of cellular identities and for acquisition of an ever more differentiated and specialized phenotype (2). Epigenetic changes are highly regulated to maintain the stability of the epigenome and cellular homeostasis. However, aberrant patterns of DNA methylation, histone modifications (acetylation, methylation, phosphorylation, etc.) and dysregulation of non-coding RNAs correlate with the development of various kinds of cancers by inactivating tumor suppressor genes, by perturbing DNA repair and chromatin remodeling, or by promoting oncogenic pathways (2, 3). These modifications are under the control of interconnected regulators. For instance, many micro-RNAs (miRNAs) can stimulate cellular proliferation by directly interacting with cell-cycle components, as this has been reported for miR-17-92, miR-221/222, miR-663, miR-302 or miR-24, which target the transcription factor E2F1 or the cyclin dependent kinase (CDK) inhibitors p27Kip1, p21CIP1 and p16INK4a, respectively (4–8). The hypermethylation of DNA, which is associated with multiple pathologies, is characterized by the transfer of methyl groups to the position 5 of cytosine residues at CpG islands, which may be located in the promoter regions of tumor suppressive genes, thus inducing their inactivation (9). This reaction is catalyzed by a family of DNA methyltransferases encoded by four specific genes (DNMT1, DNMT2, DNMT3a and DNMT3b) that synergistically promote oncogenesis (9–11). Of note, hypermethylation of DNA is perfectly reversible, and silent genes can be reactivated by administration of hypomethylating agents. Two demethylating drugs were approved by the FDA for this purpose: 5-azacytidine and the cytidine analog 5-aza-2’-deoxycytidine also known as decitabine (sold under the brand name dacogen, DAC). After their incorporation into genomic DNA, both agents directly inhibit DNMTs. In the clinic, they are exclusively prescribed for the treatment of myelodysplasia and acute myeloid leukemia (12). However, despite promising preliminary preclinical data (such as the promotion of cancer cell apoptosis *in vitro* and the reduction of tumor growth in mouse models), 5-azacytidine and decitabine provoke considerable side-effects in patients (e.g. mutagenicity, thrombocytopenia and prolonged neutropenia), limiting their employment and motivating their continuous investigation in clinical trials (13). For this reason, the search for ever less toxic hypomethylating agents is ongoing.

Recently, local anesthetics (LA) such as bupivacaine, levobupivacaine, lidocaine, ropivacaine and procaine were described to act as non-nucleoside DNA demethylating agents responsible for upregulating transcriptionally silent genes (14–21), to interfere with the expression of several miRNAs and to impact on the level of histone acetylation (22). These LA are currently employed for their analgesic and anti-inflammatory properties, but also turned out to be endowed with potent anti-tumor effects (23–33).

### Local anesthetics induce anticancer effects

LA are commonly used during oncological surgery to relief the acute pain generated by the surgical procedure. Several retrospective clinical trials reported a notable improvement of overall survival and a reduction in recurrence after primary tumor resection under local anesthesia compared to general anesthesia alone (23, 26, 34–36). This epidemiological evidence suggests that LA might have anticancer effects. Several pathways that may explain such antineoplastic effects have been described in the literature. Indeed, preclinical data indicate that LA influence the migration and the survival of cancer cells. At clinically relevant concentrations, LA inhibit the proliferation of cancer cells by provoking cell cycle arrest, by triggering mitochondrial dysfunction or by causing apoptotic cell death (28, 29, 37). Moreover, LA abrogate the migration of cancer cells after inducing intracellular Ca2+ changes that affect the cytoskeleton (24). LA also inhibit the secretion of matrix metalloproteinases necessary for the invasion of cancer cells into the extracellular matrix (38). The anti-inflammatory property of LA reduces the levels of procarcinogenic cytokine interleukin-6 (IL-6) detectable in the serum of patients during oncological surgery (25, 39). *In vivo*, LA elicit an anticancer immune response, thus causing tumor growth reduction in mice and extending the lifespan of animals with solid tumors (20, 40). When combined with chemotherapeutic agents such as 5-fluorouracil, paclitaxel or platinum salts, LA induce a synergistic antitumor effect, meaning that they sensitize cancer cells to the cytotoxicity of chemotherapy (14, 41). Taken together, the current state of the literature supports the contention that LA may directly kill cancer cells and also promote immune responses against neoplastic cells.

Hitherto, only few prospective trials investigated the role of local anesthetics on oncological prognosis (42). Most studies failed to support a direct impact on clinical outcome. However, the continued accumulation of irrefutable preclinical data demonstrating antitumor effects of local anesthetics encourages clinicians to further pursue investigations as illustrated by several randomized controlled trials recorded at www.clinicaltrials.gov and summarized in (43). Among the published scientific readouts, it can be suspected that at least some of these effects are secondary to LA effects on the tumor epigenome. Here, we summarize preclinical data highlighting the epigenetic mode of action through which LA could exert their antineoplastic activity.

### Local anesthetics promote DNA demethylation and restore expression of tumor suppressor genes

**Figure 1:**
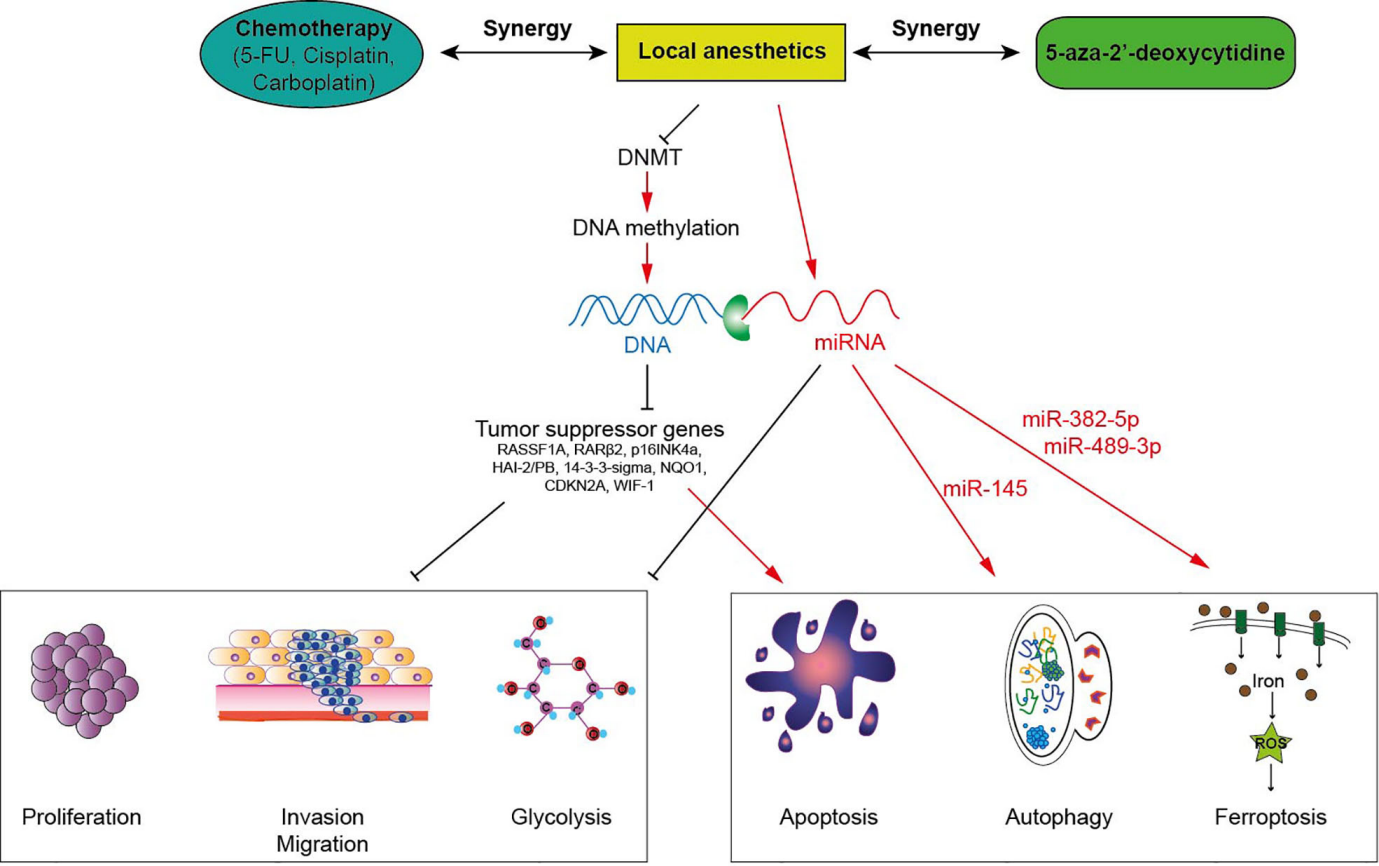
Local anesthetics induce anti-tumor effects *via* epigenetic modulation in cancer cells. Local anesthetics inhibit DNA methyltransferases (DNMT) decreasing the level of DNA methylation. This hypomethylation (or demethylation) restores the expression of various tumor suppressor genes impeding the proliferation, the invasion and the mitochondrial metabolism of tumor cells. This epigenetic effect of local anesthetics potentiates the cytotoxic activity of antineoplastic therapies.

Several studies observed that aminoamide-type local anesthetics such as bupivacaine, lidocaine, ropivacaine and ester-type local anesthetic like procaine mediate antitumor effects as well as global DNA demethylation in many types of solid cancers in a time-and dose-dependent manner (**Table 1**). For instance, bupivacaine, lidocaine and ropivacaine turned out to be potent DNA-demethylating agents of RASSF1A, hampering the proliferation of human hepatocarcinoma HepG2 and BEL-7402 cells (45). Lidocaine triggered apoptosis of human breast cancer BT-20 and MCF-7 cells by inducing the expression of the tumor suppressive RARβ2 and RASSF1A genes (14). Procaine reduced global DNA methylation by 40% in breast cancer MCF-7 cells by inhibiting DNMT1 (21) and showed an outstanding ability to minimize the growth, the proliferation and the invasion of various human cancers both *in vitro* and *in vivo* (15, 17, 20, 21). Interestingly, LA can sterically inhibit DNMT binding to CpG islands or to DNA (15, 21, 47) (**Figure 1**). As a consequence, the epigenetic regulation by LA could represent a therapeutic option. Indeed, the cytotoxic effects of conventional chemotherapeutic agents such as cisplatin or carboplatin are significantly potentiated when they are combined with LA (14, 17, 45). The association of both lidocaine and cisplatin triggers a higher level of cancer cell apoptosis than lidocaine or cisplatin alone because of the re-expression of the RASSF1A and RARβ2 genes (14). Combined with 5-aza-2’-deoxycytidine, an interesting additive demethylating effect was observed for lidocaine (44).

**Table 1:** Local anesthetics and DNA demethylation.

Agents	Cancer	Human cell lines	Epigenetic changes	Anticancer effects	Ref
Lidocaine Ropivacaine	Breast	BT-20 (estrogen receptor negative) MCF-7 (estrogen receptor positive)	Global DNA demethylation Lidocaine + 5-aza-2′-deoxycytidine induce additive demethylating effect		(44)
Lidocaine	Breast	BT-20 (estrogen receptor negative) MCF-7 (estrogen receptor positive)	Global DNA demethylation Unchanged mRNA expression of tumor suppressor genes *RASSF1A, MYOD1* and *GSTP1*		(16)
Lidocaine	Breast	MCF-7 (estrogen receptor positive) MDA-MB-231	Global DNA demethylation Demethylation of tumor suppressor genes *RARβ2* and *RASSF1A* (restoration of expression) Increased cisplatin cytotoxicity	Apoptosis	(14)
Lidocaine Ropivacaine Bupivacaine	Liver	HepG2 BEL-7402	Demethylation of tumor suppressor genes *RASSF1A* (restoration of expression) Local anesthetics + cisplatin potentiate *RASSF1A* expression	Proliferation inhibition	(45)
Procaine	Breast	MCF-7 (estrogen receptor positive)	Global DNA demethylation by inhibiting DNMT1 Demethylation of the CpG islands of the tumor suppressor gene *RARβ2* (restoration of expression)	Growth inhibition	(21)
Procaine	Liver	HLE HuH6 HuH7	Global DNA demethylation Demethylation of *p16INK4a, HAI-2/PB, 14-3-3-sigma* and *NQO1* genes (restoration of expression)	Proliferation inhibition (HLE cells) Growth inhibition (xenograft tumor)	(20)
Procaine	Colon	HCT116	Procaine alone (3µM) or combined with carboplatin (3µM) induce demethylation	Reduced viability	(17)
Procaine	Gastric	SGC-7901	Global DNA demethylation by repressing DNMT1 and DNMT3a activity Demethylation of the tumor suppressor genes *CDKN2A* and *RARβ2*	Proliferation inhibition Apoptosis	(15)
Procaine	Lung	H460 A549	Demethylation of *WIF-1* (restoration of expression)		(46)

DNMT, DNA methyltransferase; RARβ, retinoic acid receptor β; RASSF1A, Ras Association Domain Family 1A.

The effects induced by LA-mediated epigenetic modulation are not limited to the restoration of tumor suppressor gene expression but also modulate the sensitivity to pain (48) and influence the response to corticoid stress during surgery (49, 50), altogether profoundly impinging on the activity of anti-tumor effectors (49, 51). Until now, opioids have been the most commonly used analgesics for controlling acute pain. However, preclinical data indicate that opioids mediate pro-tumorigenic effects via the activation of matrix metalloproteinases and oncogenes like c-Myc as well as *via* an increase in DNA methylation (52–54). Of note, DNA methylation leads to the expression of the mu opioid receptor and predicts the response to endogenous endorphins and opioid analgesics (55). Paradoxically, excessive administration of opioids increases the risk of hyperalgesia during the postoperative period. It is tempting to speculate that the epigenetic demethylating activity of LA could prevent the hyperalgesia induced by both hypermethylation and opioids and hence counteract the opioid-mediated protumoral effects as well. Thus, opioid-free anesthesia, in which opioids are replaced by a mix of local anesthetics and other analgesic agents, offers a possibility to relieve pain, and to alleviate surgical stress-induced epigenetic changes, thereby restoring the expression of tumor suppressor genes.

### Local anesthetics regulate non-coding RNAs

**Figure 2:**
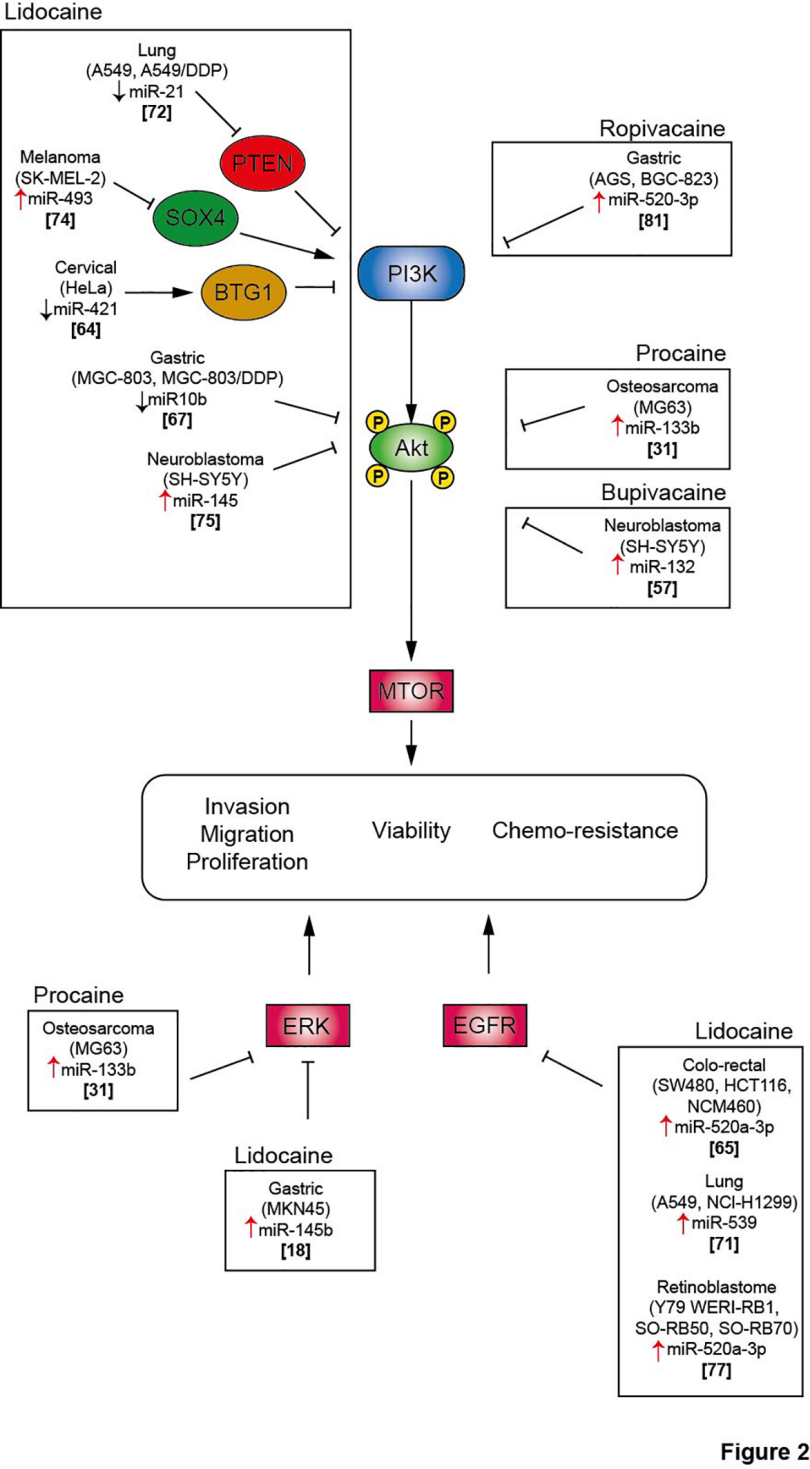
Local anesthetics inhibit cell proliferation, migration and invasion and promote cancer cell death *via* inhibition of several signaling pathway. Akt, protein kinase B; BTG1, B cell translocation gene 1; DDP, cisplatin; EGFR, Epithelial growth factor receptor; ERK, extracellular signal-regulated kinase; mTOR, mammalian Target of Rapamycin; PI3K, phosphoinositide-3 kinase; PTEN, Phosphatase and TENsin homolog; SOX4, SRY-Box Transcription Factor 4.

MiRNAs belong to the family of non-coding RNAs. Their main role is to control gene expression at different levels, and their dysregulation may trigger malignant transformation (56). LA are endowed with the capacity to enhance or suppress the expression of a variety of miRNAs, which differ according to the employed molecules and cancer cell lines (**Table 2**). The regulation of miRNAs by LA impacts several signaling pathways that mediate oncosuppression. Most of these pathways repress the downstream signaling pathway mediated by protein kinase B (PKB, best known as AKT) and mammalian target of rapamycin (mTOR), thus deeply affecting the proliferation, migration and invasion of cancer cells and inducing apoptosis (**Figures 1**, **2**) (81). Interestingly, mTOR was described as a major regulator of energy metabolism by controlling oxidative phosphorylation (84). LA are known to induce mitochondrial dysfunction leading to the production of reactive oxygen species. Indeed, the antitumor activity of ropivacaine involves both the disruption of mitochondrial function and the inhibition of Akt and mTOR phosphorylation, highlighting a putative link between AKT/mTOR and mitochondrial activity in cancer (85). Moreover, the inhibition of the AKT-mTOR pathway by LA demonstrated a relevant impact in preclinical experiments. Indeed, lidocaine-promoted miRNA regulation reversed cisplatin-resistance in MGC-803/DDP gastric cells, minimized the cisplatin resistance in lung cancer cells A549/DDP and increased the cytotoxicity of 5-fluorouracil against SK-MEL-2 melanoma cells *via* upregulation of miR-493 (67, 72, 74). LA also exert antineoplastic properties by acting on the epithelial growth factor receptor (EGFR) axis. For instance, lidocaine inhibits the proliferation of lung cancer cells *via* upregulation of miR-539, which directly targets EGFR (71). Lidocaine also minimizes the progression of retinoblastoma both *in vitro* and *in vivo* by downregulating EGFR expression through the upregulation of miR-520a-3p (77).

**Table 2:** Local anesthetics and non-coding RNAs regulation.

Agents	Cancer	Human cell lines	Epigenetic changes	Target	Anticancer effects	Ref
Bupivacaine	Neuroblastoma	SH-SY5Y	miR-132 upregulation	IGFR1 Decrease in p-Akt	Proliferation inhibition Apoptosis	(57)
Bupivacaine	Neuroblastoma	SH-SY5Y	lncRNA ZFAS1 upregulation	miR-421 downregulation ZNF564 upregulation	Apoptosis	(58)
Bupivacaine	Neuroblastoma	SH-SY5Y	lncRNA MALAT1 upregulation	miR-101-3-3p downregulation PDCD4 upregulation	Apoptosis	(59)
Bupivacaine	Neuroblastoma	SH-SY5Y	LINC00665 downregulation	hsa-miR-34a-5p	Apoptosis	(60)
Bupivacaine	Gastric	AGS HGC27	miR-145-5p upregulation	Decrease in Circ_0000376	Migration and invasion inhibition Glycolysis inhibition Apoptosis	(61)
Bupivacaine	Breast	MCF-7	miR-187-5p upregulation	lncRNA DANCR and MYB downregulation	Inhibition of migration Apoptosis	(62)
Levobupivacaine	Gastric	HGC27 SGC7901	miR-489-3p upregulation	SLC7A11	Growth inhibition Ferroptosis	(63)
Lidocaine	Breast	MCF-7	miR-187-5p upregulation	lncRNA DANCR and MYB downregulation	Migration inhibition Apoptosis	(62)
Lidocaine	Cervix	HeLa	lncRNA-MEG3 upregulation	miR-421 downregulation BTG1 upregulation	Proliferation inhibition Tumor growth inhibition Apoptosis	(64)
Lidocaine	Colon Rectum	SW480 HCT116 NCM460	miR-520a-3p upregulation	EGFR inhibition	Proliferation inhibition Apoptosis	(65)
Lidocaine	Colon Rectum	SW620 LoVo	CirclTFG2 upregulation	miR-1204 downregulation SOCS2 upregulation	Proliferation invasion and promotion inhibition Apoptosis	(66)
Lidocaine	Gastric	MGC-803 MGC-803/DDP	miR10b downregulation	AKT/mTOR inhibition	Migration and invasion inhibition Cisplatin-resistance reduction	(67)
Lidocaine	Gastric	GES-1 AGS HGC-27	Circ_ANO5 upregulation	miR-21-5p downregulation LIFR upregulation	Proliferation, migration and invasion inhibition Tumor growth inhibition Apoptosis	(68)
Lidocaine	Gastric	MKN45	miR-145 upregulation	MEK/ERK and NF-κB Inactivation	Growth, migration and invasion inhibition Apoptosis	(18)
Lidocaine	Glioma	U-251MG T98G	CircEZH2 downregulation	miR-181b-5p upregulation	Proliferation, migration and invasion inhibition Tumor growth inhibition	(69)
Lidocaine	Liver	Huh7 Hep3B	Circ_ITCH upregulation	miR-421 downregulation CPEB3 upregulation	Proliferation, migration and invasion inhibition Apoptosis	(70)
Lidocaine	Lung	A549 NCI-H1299	miR-539 upregulation	EGFR inhibition	Migration and invasion inhibition Apoptosis	(71)
Lidocaine	Lung	A549 A549/DDP	miR-21 downregulation	PTEN/PI3K/AKT PDCD4/JNK	Migration and invasion inhibition Apoptosis	(72)
Lidocaine	Lung	A549 PC9	Circ_PDZD8 downregulation	miR-516b-5p upregulation GOLT1A downregulation	Apoptosis	(73)
Lidocaine	Melanoma	SK-MEL-2	miR-493 upregulation	Sox4 downregulation Decrease in p-PI3K, p-AKT, p-Smad2	Apoptosis 5-FU cytotoxicity increase	(74)
Lidocaine	Neuroblastoma	SH-SY5Y	miR-145 upregulation	PI3K/AKT/mTOR inhibition	Growth inhibition Autophagy	(75)
Lidocaine	Neuroblastoma	SH-SY5Y	LINC01347 downregulation	hsa-miR-145-5p upregulation	Apoptosis	(76)
Lidocaine	Ovary Breast	SKOV-3 T47D	miR-382-5p upregulation	SLC7A11 downregulation	Proliferation, migration and invasion inhibition Tumor growth inhibition Reactive Oxygen Species production Ferroptosis	(19)
Lidocaine	Retinoblastoma	Y79 WERI-RB1 SO-RB50 SO-RB70	miR-520a-3p upregulation	EGFR inhibition	Proliferation inhibition Apoptosis	(77)
Lidocaine	Skin	A431	miR-30c upregulation	SIRT1 downregulation	Proliferation inhibition Inhibition of cisplatin resistance	(6)
Procaine	Osteosarcoma	MG63	miR-133b upregulation	Decrease in p/t-AKT, p/t-ERK, and p/t-S6	Proliferation and migration inhibition Apoptosis	(31)
Ropivacaine	Breast	MCF-7 MDA-MB-231	miR-27b-3p upregulation	YAP downregulation	Proliferation, migration and invasion inhibition Tumor growth inhibition Apoptosis	(78)
Ropivacaine	Cervix	Siha Caski	miR-96 downregulation	MEG2 upregulation	Growth inhibition Apoptosis	(79)
Ropivacaine	Choriocarcinoma	NA	LNCOGFRP1 downregulation	miR-4731-5p upregulation HIF3A downregulation	Viability, migration and invasion inhibition	(80)
Ropivacaine	Gastric	AGS BGC-823	miR-520a-3p upregulation	PI3K/AKT inhibition	Proliferation, migration and invasion inhibition Apoptosis	(81)
Ropivacaine	Glioma	T98G LN229	circSCAF11 downregulation	miR-145-5p upregulation	Proliferation, migration and invasion inhibition Tumor growth inhibition Reactive Oxygen Species Apoptosis	(30)
Ropivacaine	Glioma	T98G LN229	SNHG16 downregulation	miR-424-5 upregulation	Proliferation, migration and invasion inhibition Apoptosis	(82)
Ropivacaine	Glioma	U87 U373 U251	miR-21-5p upregulation	KANSL2 downregulation	Proliferation, migration and invasion inhibition Apoptosis	(83)

The extracellular signal-regulated kinases (ERK) signaling pathway is also impacted by the modulation of miRNA expression induced by LA. In a model of osteosarcoma, procaine significantly blocked the proliferation and migration of tumor cells and promoted apoptosis by upregulating miR-133b. In parallel, the level of p/t-ERK was profoundly decreased. The employment of miR-133b inhibitors reversed all the observed effects including the phosphorylation of ERK, revealing the interaction between this pathway and non-coding RNAs (31). Interestingly, the regulation of miRNAs by LA can target several pathways, thus inducing synergistic effect. Thus, lidocaine can upregulate the expression of miR-145b, which simultaneously inactivates both ERK and NF-κB pathways, potentiating the inhibition of proliferation, migration and invasion of malignant gastric cells (18).

Interestingly, different modalities of cell death triggered by epigenetic modulation were observed after LA treatment. The upregulation of miR-145 by lidocaine promoted autophagic flux in neuroblastoma SH-SY5Y cells (75). Lidocaine and levobupivacaine both induced ferroptosis by upregulating miR-382-5p and miR-489-3p, respectively (19, 63). The impact of LA on cellular stress and death pathways *via* the control of non-coding RNA emphasizes the possibility to use LA as novel antineoplastic therapeutics.

Finally, several reports suggest an intertwined regulation of multiple non-coding RNAs by LA. Indeed, lncRNAs and circular RNAs (circRNAs), a group of non-coding RNAs described to be involved in oncogenesis, may act as miRNA sponges. In a model of glioma, the treatment with ropivacaine suppressed tumor progression by upregulating the circRNA circSCAF11, while downregulating miR-145-5p (30). Inversely, bupivacaine decreased the expression of circ_0000376 while enhancing miR-145-5p in gastric cancer cells (61). Lidocaine hampered the proliferation of colorectal cancer cells by upregulating circlTFG2 and then decreasing miR-1204 (66). In a model of gastric cancer, lidocaine hindered tumor progression by modulating the miR-21-5p/LIFR axis *via* the overexpression of circ-ANO5 (68). Bupivacaine impeded neuroblastoma progression by modifying the expression of various long non-coding RNAs (ZFAS1, MALAT1, LINC00665, which sponged protumorigenic miR-421, miR-101-3-3p and miR-34a-5p, respectively) (58–60).

### Local anesthetics repress histone acetylation in cancer cells

Previous publications reported that levobupivacaine, an amino amide LA widely used to control acute surgical pain, possesses the capacity to attenuate the oncological properties of several cancer types (86, 87). However, the mechanisms by which levobupivacaine exerts its anticancer activity remain poorly characterized. Lysine acetyltransferase 5 (KAT5) acetylates both non-histone and histone proteins and increases the invasiveness of cancer cells (88). Levobupivacaine inhibits the expression of KAT5 in osteosarcoma cells, thus inhibiting their proliferation and limiting their survival (22). This preclinical finding demonstrated the implication of LA in epigenetic changes on histones leading to anticancer properties. Interestingly, the inhibition of histone acetyltransferase activity decreases opioid-induced hyperalgesia in mice (89). Nevertheless, the impact of LA on histone modification as well as the oncological consequences remain unclear, calling for future exploration.

## Discussion

The reversal of cancer-associated epigenetic dysregulations represents one possible antineoplastic strategy. Various demethylating molecules were characterized at the preclinical level (as exemplified by curcumin, (−)-epigallocatechin-3-gallate, N-phthalyl-tryptophan and zebularine) (90–94), and two agents (5-azacytidine and decitabine) have been approved by the FDA and EMA to treat patients with myelodysplastic syndrome or acute myeloid leukemia. These agents inhibit DNMT and hence reduce the global DNA methylation level in cancer cells. Despite their established anti-tumor activity, 5-azacytidine and decitabine induce severe myelosuppression, thus calling for the identification of novel epigenetic modulators.

Surprisingly, LA mediate significant antineoplastic activities by directly killing cancer cells and indirectly by eliciting anticancer immune responses (27, 32, 33, 37, 79, 95, 96). The detailed molecular comprehension of these effects may open a novel era in onco-anesthesia. Notably, the discovery of LA-promoted antitumor effects involving the induction of apoptosis secondary to the reduction of DNA methylation or the modulation of miRNAs has spurred much interest (18, 20, 30, 31, 67). Both amide and ester-type local anesthetics reduce global methylation levels in the promoter regions of tumor suppressor genes as a result of the inhibited interaction of DNMT with DNA. However, most preclinical studies have not yet investigated the effects of LA on the methylation of promoters of specific tumor suppressor genes as well as on the mRNA expression of such genes.

Beyond their effects on DNA methylation, LA also modulate (enhance or reduce) the expression of miRNAs in cancer cells, as summarized in a previous review (97). Compared to this published work, our review is the first one to critically evaluate all epigenetic changes induced by LA, including demethylating effects as well as miRNA regulation and histone acetylation, and to discuss their putative synergistic interaction with 5-azacytidine, decitabine and cytotoxicants. We surmise that the epigenetic effects of LA could be clinically relevant. Indeed, LA are well-known analgesics with a favorable toxicological profile that are commonly used during oncological intervention. A positive clinical impact of LA on cancer recurrence would provide a low-risk and low-cost benefit to oncological patients. However, before such a conclusion can be reached, further clinical and translational research must confirm the capacity of LA to improve the outcome of surgical procedures, especially if they are preceded or followed by (neo)adjuvant chemotherapy or immunotherapy. It will be particularly important to investigate the short-term (intra-operational) and long-term (post-operational) effects of LA on epigenetic signatures including DNA methylation patterns and the expression of non-coding RNAs in further translational studies.

## Author contributions

LB, OK and GK wrote the manuscript. All authors listed have made a substantial, direct, and intellectual contribution to the work and approved it for publication.

## Funding

OK is supported by Institut National du Cancer (INCa) and the DIM Elicit of the Ile-de-France. LB received a research grant by Bristol Myers Squibb Foundation France. GK is supported by the Ligue contre le Cancer (équipe labellisée); Agence National de la Recherche (ANR) – Projets blancs; AMMICa US23/CNRS UMS3655; Association pour la recherche sur le cancer (ARC); Association “Ruban Rose”; Cancéropôle Ile-de-France; Fondation pour la Recherche Médicale (FRM); a donation by Elior; Equipex Onco-Pheno-Screen; European Joint Programme on Rare Diseases (EJPRD); Gustave Roussy Odyssea, the European Union Horizon 2020 Projects Oncobiome and Crimson; Fondation Carrefour; INCa; Inserm (HTE); Institut Universitaire de France; LabEx Immuno-Oncology (ANR-18-IDEX-0001); the Leducq Foundation; a Cancer Research ASPIRE Award from the Mark Foundation; the RHU Torino Lumière; Seerave Foundation; SIRIC Stratified Oncology Cell DNA Repair and Tumor Immune Elimination (SOCRATE); and SIRIC Cancer Research and Personalized Medicine (CARPEM). This study contributes to the IdEx Université de Paris ANR-18-IDEX-0001.

## Acknowledgments

The authors are grateful to the support of Gustave Roussy Cancer Campus, Université Paris-Saclay.

## Conflict of interest

OK is scientific co-founder of Samsara Therapeutics. GK has been holding research contracts with Daiichi Sankyo, Eleor, Kaleido, Lytix Pharma, Osasuna, PharmaMar, Samsara, Sanofi, Sotio, Vascage and Vasculox/Tioma. GK is on the Board of Directors of the Bristol Myers Squibb Foundation France. GK is a scientific co-founder of everImmune, Osasuna Therapeutics, Samsara Therapeutics and Therafast Bio. GK is the inventor of patents covering therapeutic targeting of aging, cancer, cystic fibrosis and metabolic disorders.

The remaining author declares that the research was conducted in the absence of any commercial or financial relationships that could be construed as a potential conflict of interest.

## Publisher’s note

All claims expressed in this article are solely those of the authors and do not necessarily represent those of their affiliated organizations, or those of the publisher, the editors and the reviewers. Any product that may be evaluated in this article, or claim that may be made by its manufacturer, is not guaranteed or endorsed by the publisher.

## Abbreviations

Ca^2+^, calcium ion; DAC, dacogen (decitabine); DNMT, DNA methyltransferase; EGFR, Epithelial Growth Factor Receptor; EMA, European Medicines Agency; ERK, Extracellular signal-Regulated Kinases; FDA, Food and Drug Administration; 5-FU, 5 fluorouracil; IL, interleukin; LA, local anesthetics; mTOR, mammalian target of rapamycin; NK, natural killer cells; RARβ, retinoic acid receptor β; RASSF1A, Ras association domain family 1A.
